# Nutritional Content and Characteristics of Pumpkin Cream Soup with Tempeh Addition as Supplementary Food for Elderly

**DOI:** 10.1155/2021/6976357

**Published:** 2021-08-16

**Authors:** Budi Setiawan, Salma S. Aulia, Tiurma Sinaga, Ahmad Sulaeman

**Affiliations:** ^1^Department of Community Nutrition, Faculty of Human Ecology, IPB University, 16680, Indonesia; ^2^Nutrition Study Program, Graduate School, IPB University, 16680, Indonesia

## Abstract

An increase in the number of elderly people indicates a higher life expectancy. However, this is also a new challenge since the elderly tends to have age-related diseases, thus the physical, psychological, and sensory disorders that will affect their nutritional status. The development of geriatric foods such as cream soup made from pumpkin and tempeh is considered to be the solution to prevent this situation. This study used a factorial randomized design, containing processing methods (fresh and instant) and the addition of tempeh (0%, 75%, and 100%). Sensory evaluation (rating and ranking test), physical characteristics (pH, yield, rehydration, and viscosity), nutritional analysis (proximate, crude fibre, dietary fibre, vitamins B6 and B12, and *β*-carotene content), and acceptance analysis of cream soup fresh and instant were examined. Physical characterization revealed that the product had a pH of 5.4–5.7, a viscosity of 1250–2190 cP, a rehydration ratio of 5.51–6.47 g mL^−1^, and a yield of 19.44%–26.9%. The result of sensory evaluation showed that the processing method and tempeh addition had a significant effect (*p* < 0.05) on the product acceptance. This also affects the nutritional value, in which fresh products had higher nutritional value than the instant product, and products with tempeh had higher ash, protein, dietary fibre, and vitamin B12 than products without tempeh. Based on this analysis, the instant cream soup with 75% tempeh is the best formula. In one portion size (50 g), instant cream soup with 75% tempeh met 10% or more of the Indonesian recommended dietary allowance (RDA) for the elderly in terms of protein, carbohydrates, fat, energy, dietary fibre, vitamin B12, vitamin B6, and vitamin A, so it can be recommended as a complementary food for the elderly.

## 1. Introduction

Nowadays, the proportion of elderly is increasing rapidly, especially in the developed countries. Around the world, 1.5 billion people are predicted to be elderly in 2050 [1]. In Indonesia, the number of elderly is about 25.64 million, accounting for 9.6% of Indonesia's population. This situation indicates that the life expectancy continues to increase into 73 years old. However, it may become a new challenge [2].

Elderly people tend to be associated with sensorial, physical, and psychological changes and age-related diseases that may cause malnutrition. Malnutrition, particularly macronutrient and micronutrient deficiencies, is common among older adults. Macronutrient deficiency including protein and energy deficiencies in community-dwelling elderly is estimated at approximately 2% and 16%, respectively [3]. A study in Sleman, Yogyakarta, informed that there were 81.86% elderly who had protein deficiency and 74.52% elderly who had energy deficiency [4]. Meanwhile the deficiency of vitamins and minerals can reach 35% [3]. Another study reported that 71.9% elderly had B12 deficiency in Bantul, Yogyakarta [5]. Vitamin A, or as one of the pro-vitamin A: *α*-carotene and *β*-carotene; vitamin B12; and vitamin B6 deficiency frequently occurs in people over the age of 75 years and is related to cognitive impairment [6–8].

In order to prevent malnutrition, the elderly require special treatments, especially in nutrition attainment. One of the solutions to address their nutritional need is the development of supplementary food. Cream soup is a type of food that is suitable for the elderly because of its smooth texture and ease of consumption. Thus, cream soup is great to be developed into geriatric food [9]. Cream soup can be formulated from various food ingredients, including pumpkin and tempeh. Pumpkin (*Cucurbita moschata*) is one functional vegetable which is also used as a fruit and has therapeutic and medicinal properties. In addition, pumpkin is a vegetable that is easy to grow, so it is inexpensive [10]. Pumpkin contains plenty of nutrients and is rich in phenolics, flavonoids, vitamins (*β*-carotene, vitamin A, vitamin C, and *α*-tocopherol), amino acids, and carbohydrates [11]. According to de Carvalho et al. [12], pumpkin contains total carotenoids ranged between 234.21 and 404.98 *μ*g g^−1^, *α*-carotene of 67.06–72.99 *μ*g g^−1^, and *β*-carotene of 141.95–244.22 *μ*g g^−1^. High *β*-carotene intake is related to cognitive function based on dementia screening in older adults [13].

Tempeh is a typical and well-known local food in Indonesia, being a source of plant proteins formed through the fermentation process of soybean by *Rhizopus* sp. [14]. This process also increases the digestibility of tempeh, due to the hydrolysis of protein into peptides during fermentation [15]. The fermentation process in tempeh generates an increase in free amino acids which leads to gain in the digestibility and bioavailability of vitamins, minerals, amino acids, proteins, and phytochemicals and, additionally, reduces antinutrient substances [15]. Watanabe [16] and the USDA [17] reported that tempeh contains vitamin B6 and vitamin B12. Vitamins B6 and B12 and folate can affect cognitive function in the elderly through the methylation system and homocysteine level [18] However, behind these benefits, fresh tempeh lasts only 1–3 days, so additional treatments are necessary to increase the shelf life of tempeh [19].

Based on the benefits of pumpkin and tempeh for the elderly's health, the development of pumpkin cream soup with the addition of tempeh was considered as an excellent option. The purpose of this study was to analyse sensory properties, physical characteristics, nutritional content, contribution to nutrient needs, and acceptance levels of cream soup by the elderly.

## 2. Materials and Methods

### 2.1. Study Design and Cream Soup Formulation

This study was an experimental research using factorial random design that combines the type of processing cream soup (fresh and instant) and the addition of tempeh (0%, 75%, and 100% of pumpkin composition) to produce six formulations, namely, A1B0 (fresh cream soup with 0% tempeh), A1B1 (fresh cream soup with 75% tempeh), A1B2 (fresh cream soup with 100% tempeh), A2B0 (instant cream soup with 0% tempeh), A2B1 (instant cream soup with 75% tempeh), and A2B2 (instant cream soup with 100% tempeh). The cream soup formula referred to Indonesia's standard for cream soup.  Table 1 presented the ingredients in all formulas.

### 2.2. Cream Soup Materials and Production

Pumpkins (*Cucurbita moschata* variety) were obtained from Pasar Anyar (local market) in Bogor and tempeh (*Grobogan* variety) were obtained from Rumah Tempe Indonesia, Bogor. The other ingredients for soup, such as carrots, leeks, onions, rice flour (for instant cream soup only), and prepared cream, were purchased at local markets in Bogor, Indonesia.

The procedure of pumpkin cream soup production was the pumpkin was cut and cleaned from the seeds then sliced into cubes, while tempeh and carrots were cut into cubes, along with tiny slices for other ingredients. The onions were sautéed and the stock was poured. The pumpkin, tempeh, carrot, and leek were added. Then, the soup was boiled for 15 minutes. After the soup was cooked, it was mixed using a blender to become a puree. For fresh cream, cooking cream was added into the puree until mixed well, while for instant cream soup, rice flour was added after the cooking cream. Furthermore, the puree was dried using a drum dyer (120°C) for 60 seconds to produce cream soup powder. The fresh cream soup was dried in the oven at 60°C for 24 hours in order to analyse the immediate and dietary fibre content, while instant cream soup powder can be used directly for the entire analysis [20].

### 2.3. Sensory Analysis

Sensory evaluation was applied on six cream soup formulas using a rating test and a ranking test out of 35 semitrained panelists. The panelists signed informed consent before the test. Rating tests were conducted by presenting the samples one by one (A1B0, A1B1, A1B2, A2B0, A2B1, and A2B2) in aluminum foil containers to maintain the temperature (60°C) with a random three-digit code. The aim of this test was to evaluate the preference level for the sample in terms of texture, colour, aroma, taste, mouthfeel, thickness, and overall on a scale of 1 to 9 (1 for extremely dislike to 9 for extremely like). Ranking tests were performed by presenting the entire sample and asking the panelists to sort the samples on a scale of 1 to 6 (1 for the most preferred sample to 6 for the least preferred sample) attributed to texture, colour, aroma, taste, mouthfeel, thickness, and overall [21].

### 2.4. Physical Characteristics Analysis

In terms of physical characterization, pH was measured using a pH meter (Ohaus Starter 3100, 180 USA) and yield was calculated based on the percentage of weight between the cream soup powder and the cream soup prior to drying [22]. The rehydration was calculated by rehydrating the instant cream soup related to the modified supernatant [23]. Viscosity analysis was carried out by inserting a sample (40 g) with 200 mL boiling water into a viscometer [24].

### 2.5. Proximate Analysis

Moisture content was determined by drying the samples in an oven at 105°C to a constant weight for about three hours. The ash content was determined by incinerating a sample in a muffle furnace at 550°C for five hours (Method No. 930.05). Protein content was conducted using the Kjeldahl method (Method No. 978.04). Meanwhile, fat content was determined by the Soxhlet extract method (Method No. 930.09) [22]. Carbohydrates were obtained by a *different* method between the whole sample and the sum of moisture, ash, protein, and fat composition of the sample The crude fibre was analysed using H_2_SO_4_ and NaOH dissolution, calculated by comparing the final weight of samples against the residual samples (Method no. 962.09). All measurements were duplicated three times [25].

### 2.6. Dietary Fibre Analysis

Dietary fibre was analysed based on the Asp et al. [26] method as a fat-free sample was analysed using terminal enzymes, pepsin, and pancreatin. Filtrate results and sample residues were analysed to determine the insoluble, soluble, and total fibres.

### 2.7. Vitamin B6 and B12 Analyses

Vitamins B6 and B12 were analysed according to the Keller [27] method with the High-Performance Liquid Chromatography-Reverse Phase (HPLC-RP). HPLC was adjusted on a C-18 column with 250 mm length and inner diameter of 4 mm as a stationary phase. The mobile phase used the standard B6 solution by preparing 100 mg standard reference into 100 mL flask. Samples were added with 60 mL of 2% acetic acid, then 25 mL methanol and 2% acetic acid were added until reaching the limit. As for the mobile phase, the analysis of vitamin B12 used A (625 mL) and B solutions (375 mL). The A solution was formulated from 0.96 g pentane sulfonic acid dissolved in 20 mL of acetic acid in 1 L volumetric flask added with 25% methanol to the limit. The B solution was made from 1.1 g heptane sulfonic acid sodium salt in 1 L flask added with 20 mL acetic acid and 25% methanol to the limit. HPLC was flashed for 3 hours then conditioned using a stationary phase followed by each mobile phase for 20 to 30 minutes until a straight baseline was obtained on the chromatogram. The sample was dissolved in a 5 mL mobile phase solution, and 20 *μ*L was injected twice with a flow rate of 0.5 mL min^−1^ at a column temperature of 25°C, a sample temperature of 10°C, and a 290 nm wavelength UV detector.

### 2.8. *β*-Carotene Analysis

This analysis used the AOAC method with HPLC-RP. HPLC was set on a C-18 column as the stationary phase, while the mobile phase used dichloromethane, methanol, and acetonitrile dichloromethane with the ratio of 200 : 300 : 100 and a flow rate of 0.5 mL min^−1^ over a column temperature of 25°C, a sample temperature of 10°C, and the 450 nm wavelength diode array detector. HPLC was flashed for 3 hours and conditioned using the stationary phase followed by each mobile phase for 20 to 30 minutes until a straight baseline was obtained on the chromatogram. The sample was dissolved in a 5 mL mobile phase solution, and 20 *μ*L was injected twice. The analysis results were obtained on the chromatogram [22].

### 2.9. Acceptance Analysis

The acceptance analysis was applied to one formula with a good sensory value and high nutritional value. Acceptance analysis was performed by a hedonic test attributed to colour, aroma, texture, taste, mouthfeel, serving temperature, thickness, and overall, with a scale of 1 to 5, where the acceptance level was assessed by serving a bowl containing cream soup (25 g and 125 mL water) within 15 to 20 minutes, then the remaining cream soup was assessed (0 to 100%; 100% for less preferred while 0% for the most preferred acceptance). The test was conducted at five integrated health centres (*Indonesian*: *Posbindu*) in Dramaga, Bogor, using inclusion criteria as the subjects were elderly at 60–95 years old.

### 2.10. Statistical Analysis

Sensory data, physical characteristics, and nutrient content were analysed using ANOVA test (*p* < 0.05) with Duncan's multiple range test, while acceptance data were analysed using the percentage and descriptive method.

## 3. Results and Discussion

### 3.1. Sensory Properties

Sensory properties are important criteria that must be met in the development and acceptance of food products [11]. For fresh cream soup, the sensory analysis showed that A1B2 had the highest value on thickness, colour, and texture parameter, while the highest value of aroma, taste, and mouthfeel was obtained in A1B0. In instant cream soup, A1B2 comprised the highest value of thickness, texture, and taste parameters, while the highest colour, aroma, and taste were found on A2B0 (Table 2). The results showed that fresh cream soup without the addition of tempeh had the highest acceptance rate. Meanwhile, instant cream soup with the addition of 75% tempeh remained acceptable for the panelists. The addition of too much tempeh (100% treatment) tended to be disliked because it caused the paler colour, created a bitter taste, and created an unpleasant aroma. Nout and Kiers [28] explained that the white colour of tempeh was caused by spore germination and growth of the mycelium of *Rhizopus* spp. Meanwhile, Kustyawati et al. [29] explained that the bitter taste and the unpleasant aroma of tempeh were attributed to the formation of hydrophobic peptides by the degradation of soy protein due to the activity of *Rhizopus* spp. Besides the addition of tempeh, the drying process also affected the sensory characteristics of cream soup. The high temperature could reduce the flavour and aroma of the product [30]. Consequently, the instant cream soup had a low acceptance rate, presumably due to the high temperature of the drying process with a drum dyer.

### 3.2. Physical Characteristics

The physical characteristics of the cream soup are presented in Table 3. The physical characterization of the whole cream soup formulas demonstrated that the pH value was influenced by the drying process. The drying process (drying method and time) could also alter the pH of the product due to changes in water content [31]. Moreover, viscosity is an important parameter in liquid foods [32]. The results showed that the viscosity of the cream soup formula ranged between 1250 and 2190 cP. This value was comparable to the viscosity of sweet potato cream soup (621.25–2380 cP) [33] but lower than that of the commercial corn cream soup (3337–3823 cP) [34]. This test also showed that fresh cream soup had a lower viscosity than the instant cream soup (*p* < 0.05). This difference was influenced by processing factors, especially the addition of rice flour. Kemashalini et al. [35] declared that the maximum viscosity of rice flour solution is between 6660 and 7400 mPas. This value was influenced by the amylose/amylopectin ratio, the fine structure of amylopectin, the length of amylose chain, and the crystallinity of each rice variety. Other physical analyses, such as product yield and rehydration ratio (only in instant cream soup), showed that the A2B0 formula had the highest yield which was expected to be generated by the addition of rice flour. According to Sudarsan et al. [36], high yield of the product will increase its economic value but does not guarantee the quality because the yield depends on the volume of swelling when the powder is rehydrated. Meanwhile, the rehydration ratio was influenced by the starch structure of the rice flour. The rehydration ratio is one of the parameters for determining the quality index, in which this parameter can reflect physical and chemical changes during dehydration [37]. Nwokocha and Williams [38] stated that the high water-binding capacity of a product is due to a starch polymer structure, whereas low water-binding capacity indicates structural cohesion. Kemashalini et al. [35] added that the molecular structure of amylose and amylopectin also influences the rehydration ratio. According to those authors, flour derived from low amylose rice, especially with a high amount of damaged starch, could increase water absorption and thereby increase the flowering power of low amylose rice flour.

### 3.3. Proximate Nutrient Content

The results revealed significant differences among the cream soup formulas in all parameters (Table 4). Analysis of water and ash content showed significant differences between fresh and instant formulas, in which fresh cream soup has higher water content than instant cream soup. The difference in water content was influenced by the drying treatment, which could reduce up to 87.7% of water content of the product [39]. Meanwhile, the ash content was affected by the transformation process, in which the heating process at a high temperature could reduce the ash content [40]. Analysis of protein and fat showed that the formula with the addition of tempeh had higher protein (21.42-31.85) and lower fat (13.30-16.46) than the formula without tempeh (9.76-10.14; 29.53-30.01). The high protein content was associated with the addition of tempeh. Based on USDA [41], tempeh contains 19 g of protein per 100 g serving or 38% daily value. Protein in tempeh had the advantage that it is easier to digest than soy protein due to the activity of the protease enzyme produced by *Rhizopus* spp. during fermentation [42]. Moreover, tempeh also has lower fat content. Vital et al. [42] mentioned that tempeh could be used as a low-fat dietary preference. Meanwhile, the higher fat content in the formula without tempeh caused the slightly higher composition palm oil in this formula. Palm oil was used to improve the aroma of the spice when it was sautéed. Palm oil contains 50% saturated fatty acids, 40% monounsaturated fatty acids, and 10% polyunsaturated fatty acids [43]. The crude fibre test showed that all cream soup formulas had higher fibre content than pumpkin pulp fibre with 3.72–10.88 g kg^−1^ [44]. The proximate content and sensory evaluation result were considered for the analysis of dietary fibre, vitamin B12, vitamin B6, and *β*-carotene. According to this analysis, A1B1 and A2B1 had good sensory and relatively high proximate content, while A1B0 and A2B0 formulas were further tested as controls.

### 3.4. Dietary Fibre Content

The results of dietary fibre content analysis showed that the soluble fibre content ranged from 7.0 to 13.4%, insoluble fibre ranged from 10.9 to 20.3%, and the total dietary fibre was between 18.1 and 30.4% (Table 5). The fibre content of the formulations with tempeh (A1B1 and A2B1) increased significantly (28.15% and 30.43%, respectively) compared to the formulations without tempeh (A1B0 (18.02%) and A2B0 (24.92%)). This result was due to the addition of tempeh, which contained a total fibre of 18.96 g 100 g^−1^ comprised of 2.18 g 100 g^−1^ soluble fibre and 16.80 g 100 g^−1^ insoluble fibre [41]. The increase in fibre content was also attributable to pumpkin usage. Kulaitienė et al. [45] reported that pumpkin is an excellent source of fibre with a maximum soluble fibre content of up to 26.50% of fresh weight. This result showed that all cream soup formulas also fulfilled the dietary fibre source standard for the Indonesia food labelling standard (ALG) with a minimum composition of 1.5 g 100 g^−1^ [46].

### 3.5. Vitamin B6 and B12 and *β-*Carotene Content

Analysis of the content of vitamins B12 and B6 revealed different results. The content of vitamin B12 differed significantly between treatments, while there was no significant difference between formulas in vitamin B6 (Table 6). The significant difference in vitamin B12 levels was thought to be caused by the addition of tempeh, in which tempeh contains vitamin B12 of 0.7–8.0 *μ*g 100 g^−1^ [16]. Vitamin B12 content of tempeh resulted from synthesis by *Klebsiella pneumoniae* and *Propionibacterium freudenreichii* during the fermentation process [47]. Analysis of *β*-carotene showed that the fresh cream soup formula (A1B0) had the highest content level, while the instant cream soup formula (A2B1) had the lowest content. The low *β*-carotene content in the instant formula was caused by the cooking and drying process. Piyarach et al. [48] stated that more than 50% of *β*-carotene, *α*-carotene, and lutein content could be degraded during the drying process. The larger surface area contributed to the lower carotenoid content [35]. Koh and Loh [49] also reported that heating, boiling, steaming, or frying reduces the *β*-carotene content. Based on this result, four formulas met the requirements for vitamin B12 and *β*-carotene, namely, vitamin B12 0.72 *μ*g and vitamin A 600 *μ*g.

### 3.6. Acceptance of the Best Formula Cream Soup Products for the Elderly

**Figure 1:**
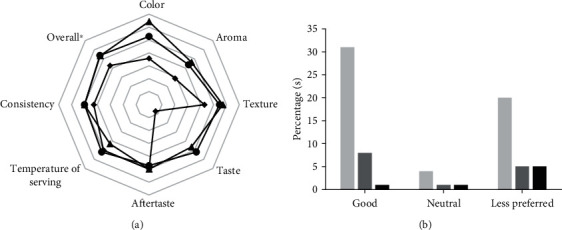
The level of preference based on preference (a) and acceptance (b) of the elderly towards cream soup: (●) youngest-old, (▲) middle-old (⬥) oldest-old, (light gray square) youngest-old, (dark gray square) middle-old, and (■) oldest-old.

The best formula for this study was the A2B1 formula for its high nutritional content with a relatively good preference level over other formulas. The subjects in this test were aged 60–95 years, including 55 youngest-old (60–69 years), 14 middle-old (70–79 years), and 6 oldest-old (≥80 years) of 8 males and 67 females. In the overall preference level, the youngest-old and middle-old have higher scores than the oldest old. Furthermore, in the taste parameter score, the oldest-old have a different result compared to the youngest-old and the middle-old. The oldest-old had a lower score than the youngest-old and middle-old (Figure 1(a)). The study reported that decreasing smell and taste were highly prevalent in the elderly [50]. The elderly had lower perception in sweet and sour flavours than adult [51].

Based on the data, the acceptance level for consuming one serving size product is about 56.36% in the youngest-old, 57.14% in the middle-old, and 14.28% in the oldest-old. The oldest-old had a lower acceptance rate than the youngest-old and middle-old (Figure 1(b)). Reduced appetite could have led to decreasing food and nutrient intake in increasing age. Moreover, changing physiology of an older body and changing psychological function can cause the acceptance level in oldest-old. It is related with their argument that they feel full after breakfast until snack time [52].

## 4. Conclusions

This study concluded that the processing type and tempeh addition in producing pumpkin cream soup affected the sensory value and nutritional content. Pumpkin cream soup with tempeh addition has an additional source of protein, along with high fibre, vitamin B12, and vitamin A for the elderly. Cream soup fulfilled 10% of Indonesian RDA for energy, fat, and fibre and can be well received as a snack by the elderly. The best formulation is instant pumpkin cream soup with 75% tempeh addition. Furthermore, clinical trials are required to determine the effect of applying cream soup on biochemical biomarkers for the elderly. Pumpkin cream soup with tempeh addition can be used as a supplementary food in a nutrition status improvement program for the elderly.

## Figures and Tables

**Table 1:** Ingredient's composition in pumpkin cream soup formula in one recipe.

Ingredients (g)	A1B0	A1B1	A1B2	A2B0	A2B1	A2B2
Pumpkin	60	50	50	60	50	50
Tempeh	0	37.5	50	0	37.5	50
Carrot	30	25	25	30	25	25
Onion	20	20	20	20	20	20
Palm oil	7	4	4	7	4	4
Cooking cream	15	15	15	15	15	15
Rice flour	0	0	0	90	30	30
Stock	200	200	200	200	200	200

**Table 2:** Sensory properties of cream soup formulas.

F	Sensory properties						
	Consistency	Colour	Aroma	Texture	Taste	Mouthfeel	Overall
A1B0	6.03 ± 1.42^bc^	6.14 ± 1.42^bc^	6.17 ± 1.32^c^	5.71 ± 1.4^abc^	5.03 ± 1.52^c^	5.40 ± 1.58^c^	5.67 ± 1.28^c^
A1B1	5.94 ± 1.30^bc^	6.49 ± 1.04^c^	5.31 ± 1.10^ab^	5.91 ± 1.31^bc^	4.14 ± 1.24^ab^	4.63 ± 1.37^ab^	4.83 ± 1.32^b^
A1B2	6.37 ± 1.26^c^	6.60 ± 1.17^c^	5.91 ± 1.27^bc^	6.17 ± 1.12^c^	4.77 ± 1.39^bc^	4.94 ± 1.35^bc^	5.28 ± 1.40^bc^
A2B0	4.71 ± 1.62^a^	5.97 ± 1.36^bc^	5.80 ± 0.93^bc^	5.17 ± 1.36^a^	4.68 ± 1.21^bc^	5.11 ± 1.16^bc^	5.06 ± 1.13^bc^
A2B1	5.86 ± 1.14^bc^	5.69 ± 1.28^b^	5.46 ± 1.24^b^	5.40 ± 1.26^ab^	4.80 ± 1.16^c^	4.89 ± 0.99^bc^	5.06 ± 1.08^bc^
A2B2	5.57 ± 1.42^b^	4.77 ± 1.26^a^	4.77 ± 1.28^a^	5.29 ± 1.45^ab^	3.77 ± 1.09^a^	4.06 ± 1.21^a^	4.17 ± 1.10^a^

Mean value ± standard deviation (SD) from three replications. The different superscripts in one column denote significant differences (*p* < 0.05).

**Table 3:** Physical properties of cream soup formulas.

F	pH	Viscosity (cP)	Rehydration power (%)	Yields (%)
A1B0	5.53 ± 0.12^ab^	1250 ± 35.35^a^	NA	NA
A1B1	5.39 ± 0.26^a^	1910 ± 14.14^ab^	NA	NA
A1B2	5.46 ± 0.12^a^	1255 ± 7.07^a^	NA	NA
A2B0	5.70 ± 0.06^c^	2190 ± 127.28^d^	6.47 ± 0.45^b^	26.90%
A2B1	5.60 ± 0.07^bc^	1560 ± 28.28^bc^	5.15 ± 0.15^a^	29.44%
A2B2	5.59 ± 0.02^bc^	1655 ± 134.35^c^	5.70 ± 0.50^a^	20.80%

F: formulas; NA: not analysed. Values are mean ± SD from three replications; the different superscripts in one column denote significant differences (*p* < 0.05).

**Table 4:** Proximate nutrient content of cream soup formulas.

F	Composition (%)					
	Moisture	Ash	Protein	Fat	Carbohydrate	Crude fibre
A1B0	7.97 ± 0.48^b^	5.72 ± 0.92^d^	10.15 ± 0.10^a^	29.53 ± 0.62^d^	46.64 ± 0.85^c^	2.85 ± 0.22^b^
A1B1	7.31 ± 0.88^b^	3.86 ± 0.05^c^	30.01 ± 0.91^d^	13.30 ± 0.60^a^	45.52 ± 0.91^b^	4.90 ± 0.15^d^
A1B2	7.12 ± 0.94^b^	4.34 ± 0.60^c^	31.85 ± 0.48^e^	15.40 ± 0.46^b^	41.22 ± 0.37^a^	5.12 ± 0.00^d^
A2B0	2.19 ± 0.53^a^	1.18 ± 0.13^a^	9.76 ± 0.32^a^	30.15 ± 0.25^d^	54.71 ± 0.37^d^	1.37 ± 0.29^a^
A2B1	1.89 ± 0.83^a^	2.06 ± 0.11^b^	21.42 ± 0.19^b^	15.47 ± 0.36^b^	59.16 ± 0.36^e^	4.20 ± 9.29^c^
A2B2	1.68 ± 0.27^a^	2.05 ± 0.15^b^	23.09 ± 0.50^c^	16.46 ± 0.59^c^	56.71 ± 0.13^d^	4.77 ± 0.19^d^

F: formulas. Values are mean ± SD from three replications; the different superscripts in one column denote significant differences (*p* < 0.05).

**Table 5:** Dietary fibre compositions of cream soup formulas.

Formulas	Soluble fibre	Insoluble fibre	Total dietary fibre
A1B0	7.06 ± 0.76^a^	10.96 ± 0.98^a^	18.02 ± 0.49^a^
A1B1	13.41 ± 0.62^c^	14.59 ± 0.39^b^	28.16 ± 0.82^c^
A2B0	10.50 ± 0.71^b^	14.81 ± 0.47^b^	24.93 ± 0.53^b^
A2B1	10.35 ± 0.91^b^	20.29 ± 0.89^c^	30.43 ± 0.44^d^

Values are mean ± SD from three replications; the different superscripts in one column denote significant differences (*p* < 0.05).

**Table 6:** Composition content of vitamin B12, vitamin B6, and *β*-carotene in cream soup formulas.

Formulas	Vitamin B12 (ppm)	Vitamin B6 (ppm)	*β*-Carotene (g kg^−1^)
A1B0	11.78 ± 0.39^c^	1.40 ± 0.08	2.48 ± 0.79^c^
A1B1	22.12 ± 0.92^d^	1.44 ± 0.06	1.89 ± 1.16^ab^
A2B0	6.04 ± 0.30^a^	1.50 ± 0.07	1.20 ± 0.11^bc^
A2B1	8.29 ± 0.04^b^	1.38 ± 0.02	0.65 ± 0.13^a^

Values are mean ± SD; the different superscripts in one column denote significant differences (*p* < 0.05).

## Data Availability

All datasets generated or analysed during this study are available upon reasonable request from the corresponding author.
